# Study of the Possible Alleviated Role of Atorvastatin on Irinotecan-Induced Lingual Mucosal Damage: Histological and Molecular Study

**DOI:** 10.1155/2021/9690047

**Published:** 2021-09-30

**Authors:** Eetmad A. Arafat, S. M. Abo El-khair, A. Z. Elsamanoudy, Dalia A. Shabaan

**Affiliations:** ^1^Histology and Cell Biology Department, Faculty of Medicine, Mansoura University, Egypt; ^2^Medical Biochemistry and Molecular Biology Department, Faculty of Medicine, Mansoura University, Egypt; ^3^Department of Clinical Biochemistry, Faculty of Medicine, King Abdulaziz University, Jeddah, Saudi Arabia

## Abstract

**Background:**

Oral mucositis is the most debilitating and troublesome adverse effect of irinotecan (CPT-11) treatment. It adversely affects the patient quality of life. The aim of this work was to study the histological, immunohistochemical, and molecular changes in the oral mucosa by CPT-11 and the possible alleviated role of atorvastatin.

**Methods:**

Rats were randomly divided into control, CPT-11-treated group, and CPT-11+ atorvastatin-treated group. At the end of the experiment, the anterior two-thirds of the tongue was dissected out and divided into two parts: one part for light microscopic examination and the second for molecular study.

**Results:**

CPT-11-treated group revealed loss of normal mucosal organization, areas of ulceration and inflammation, and loss of architecture of lingual papillae. A significant decrease in immunohistochemical and molecular gene expression of Ki-67 and antiapoptotic Bcl-2 levels was observed. A significant increase in NF-*κ*B immunohistochemical and mRNA gene expression level and a nonsignificant increase in Nrf2 gene expression were detected. Coadministration of atorvastatin showed remarkable improvement in the histopathological picture with a significant increase in Ki-67 and Bcl-2, a significant decrease in NF-*κ*B protein and gene expression, and a significant increase in Nrf2 gene expression.

**Conclusion:**

Atorvastatin substantially attenuates CPT-11-induced oral mucositis through the initiation of the antiapoptotic gene, modulation of the inflammatory, and antioxidant gene expression.

## 1. Introduction

Chemotherapy drugs are a widely used cancer treatment approach that interferes with the division of cancer cells and can be administered either alone or in combination with other modalities [1].

Chemotherapy's biggest drawback is its lack of selectivity, as it works on both tumor cells and healthy cells that proliferate quickly [2]. For example, the oral mucosa is lined with a rapidly proliferating epithelium that is highly sensitive to chemotherapy damage [3]. Oral mucosal injury is clinically important because it adversely affects a patient's quality of life [4].

Irinotecan (CPT-11) is a camptothecin derivative and a recently developed chemotherapy drug effective against multiple malignant tumours, including colorectal, stomach, ovary, and lung cancers, as well as central nervous system tumours, including recurrent glioblastomas, rhabdomyosarcomas, and other sarcomas [5]. It inhibits topoisomerase-1, an enzyme involved in DNA replication, and causes several single-strand DNA breakdowns and cell division inhibition [6].

Mucosal damage and inflammation are the most debilitating and troublesome adverse effects of CPT-11 treatment. For example, gastrointestinal mucositis is most noticeable in the oral cavity and the small intestine, and its extent depends on treatment regimen, treatment duration, patient age, and patient-related genetic factors [7]. Patients with severe mucositis that are undergoing multicycle chemotherapy or combined regimens may require delayed treatment cycles or reduced dosages [8]. Until recently, the management of oral mucositis was mainly palliative and included oral hygiene, analgesics or local anaesthetics, and protective coating agents [9].

The statin family of drugs is one of the chief drugs used in the treatment of hypercholesterolemia and coronary artery diseases. Statins also speed up epithelization and accelerate wound healing by preventing the adhesion of leukocytes onto inflammation sites to consequently decrease inflammatory cytokines. Statins have also been proven to improve macrophage accumulation at the site of injury, thus, enhancing keratinocytes and endothelial cell proliferation [10]. To date, no evidence-based approaches for the avoidance and treatment of oral mucositis exist [11]. Statin drugs may be useful against oral mucositis, but its principal mechanism of action is incompletely understood. This study investigated structural changes in oral mucosa under CPT-11 treatment and the possible role of atorvastatin in mucositis alleviation via histological, immunohistochemical, and molecular investigations.

## 2. Materials and Methods

### 2.1. Chemicals

CPT-11 was obtained from Sigma Aldrich Chemical Co (Cat # 1347609 USP Sigma Aldrich). Atorvastatin (20 mg) was purchased from Pfizer Company for Pharmaceutical and Chemical industries (New York, USA).

### 2.2. Experimental Animals

The experiment was carried out on 50 adult Sprague–Dawley male albino rats that weighed 180–200 g and were 6 weeks old. The rats were obtained from the animal house at the Faculty of Pharmacy, Mansoura University. The animals were sheltered in plastic cages (2 rats/cage) with stainless steel wire-bar lids. They were held in a controlled temperature (22°C ± 2°C) and humidity under a constant 12 h light-dark cycle. The rats were allowed free access to water and a standard rat diet during this period. The rats were acclimatized for one week before the experiment began. The experimental protocols and procedures were reviewed and permitted by the institutional review board, Faculty of Medicine, University of Mansoura (Ref R.21.04.1293).

### 2.3. Experimental Protocol

The rats were randomly allocated into three groups:

Group I (control group): 30 rats were equally divided into three subgroups of 10 rats each: subgroup Ia: negative control distilled water (-ve control DW) rats were given DW (1 mL) by gastric tube once daily for 5 days; subgroup Ib: negative control buffer (-ve control buffer) rats were given single IP injections of (sorbitol/lactic acid buffer; 45 mg/mL of sorbitol, 0.9 mg/mL of lactic acid, pH 3.4); and subgroup 1c: positive control atorvastatin (+ve control Atorvastatin) rats were given atorvastatin dissolved in DW (5 mg/kg) by gastric tube once daily for 5 days.

Group II: (CPT-11-treated group): 10 rats received 200 mg/kg of irinotecan in a sorbitol/lactic acid buffer as a single IP injection [12].

Group III: (CPT-11 + atorvastatin-treated group): 10 rats received a single IP injection of irinotecan as in group II + atorvastatin dissolved in DW and administered via a gastric tube in a dose of 5 mg/kg. The treatment occurred daily for 5 days [13].

At the end of the experiment, (24 hours from the last dose of atorvastatin) the rats (*N* = 50) were sacrificed by IP administration of pentobarbital (40 mg/kg), then the tongue of each rat was dissected out, and its anterior two-thirds was divided into two parts. The first part was fixed in 10% buffered formalin and prepared for light microscope examination. The second part was snap-frozen in liquid nitrogen and stored at −80°C for molecular study.

#### 2.3.1. Histological Study

The specimens were fixed in 10% buffered formalin, dehydrated, and processed for paraffin block preparations. The 5 *μ*m thick paraffin sections were obtained and used for light microscopic study using hematoxylin and eosin (H&E) [14].

#### 2.3.2. Immunohistochemical Staining

Multiple immunohistochemical markers were used, including Ki67, Bcl2, and nuclear factor kappa-beta (NF-*κ*B). Ki67 is a nuclear protein closely related to the cell cycle and is a marker of cell proliferation [15]. Bcl2 is an antiapoptotic marker [16]. NF-*κ*B acts as a major mediator of inflammation in a variety of inflammatory diseases [17]. Primary antibodies used in this study are Ki-67 using rabbit monoclonal [SP6] antirat Ki67 (1 : 500; Abcam); Bcl-2 Mouse monoclonal Cat. # sc-7382, dilution 1|50 Santa Cruz Biotechnology; and NF-*κ*B rabbit polyclonal antibody; Cat. #RB-9034-R7; dilution 1/100; Thermo Scientific, CA, USA.

The 5 *μ*m thick paraffin sections on positively charged glass slides were deparaffinized and hydrated by boiling the sections for 15 m in 10 mM citrate buffer for antigen retrieval. Nonspecific reactions were inhibited by putting the slides in 3% bovine serum albumin for 30 m. Next, the sections were incubated overnight with the primary antibody at 4°C. Endogenous peroxidase activity was blocked with 10% H2O2 in PBS. Detection was achieved via incubation in biotinylated secondary antibodies for 1 h, then labeled horseradish peroxidase for 1 h, followed by 3, 3′-diaminobenzidine (DAB) as a chromogen. Mayer's hematoxylin was used for counterstaining. Negative control sections were obtained by replacing the 1ry antibodies with phosphate-buffered saline. Positive immunohistochemical staining for Ki-67 was detected by brown nuclei, while Bcl2 and NF-*κ*B were identified as brown cytoplasmic staining [18].

#### 2.3.3. Morphometric Study

An optical microscope (Olympus, Tokyo, Japan) joined to a Leica digital camera (ICC50) was used for image capturing. The images were analyzed on an Intel Core I3-based computer using Video Test morphology software (Saint Petersburg, Russia). All measurements were taken at a magnification (400×). Five randomly selected, nonoverlapping fields were examined from each rat (5 rats/group) to measure:

1. The two main diameters of lingual papillae (mean length and width of the filiform and fungiform) using H&E-stained sections
2. The number of Ki-67 positive cells
3. The number of BcL2 positive cells
4. The area % of positive anti-NF*κ*B immune reactions

#### 2.3.4. Molecular Study

The liquid nitrogen frozen tongue tissue samples (30–50 mg) were utilized for total RNA extraction and the real-time qRT-PCR assessment of Ki-67, Bcl-2, NF-*κ*B, and transcription factor NF-E2-related factor (Nrf2) expression.

Total RNA was extracted from the rats' tongue via the Tri-Fast TM reagent (PeqLab. Biotechnologie GmbH, Carl-Thiersch St. 2B 91052 Erlongen, Germany, Cat. No. 30-2010), and the purity was estimated via a NanoDrop™ 2000 Spectrophotometer (Thermo Scientific, USA). The cDNA synthesis by reverse transcription reactions was performed with the Maxima First Strand cDNA Synthesis Kit (Thermo Scientific, Waltham, MA, USA, cat No. #K1641). Ki-67, Bcl-2, NF-*κ*B, and Nrf2 expressions were quantified via real-time PCR (Applied Biosystem 7500, real-time PCR detection system-Life Technology, Carlsbad, CA, USA-Applied Biosystem SYBR® Green PCR Master Mix (2X)-Life Technology, USA, Cat. no. 4344463). The thermal cycling reaction was performed as follows: the mixtures were incubated for 10 min at 95°C, then 40 cycles of 15 s at 95°C, 1 min at 60°C and, and finally, 15 s at 95°C, 1 min at 60°C, and 15 s at 95°C. The primer sequences used [19–21] were described in supplement material 1.

#### 2.3.5. Statistical Analysis

The collected data were coded, processed, and analyzed using the SPSS (Statistical Package for Social Sciences) version 26 for Windows® (IBM SPSS Inc., Chicago, IL, USA).

Quantitative data were expressed as mean ± SD (standard deviation). One-way analysis of the variance was used to test the significance between three and more independent groups. Post hoc Tukey was used to assess the significance between every two adjacent groups.

In all applied tests, the *P* values associated with test statistics indicated the significance level at which the null hypothesis (the hypothesis of no difference) was rejected, and it was set at 0.05 so that *P* values ≥ 0.05 are statistically nonsignificant, *P* values < 0.05 are significant, and *P* values < 0.01 are highly significant.

## 3. Results

### 3.1. H&E Stain

**Figure 1:**
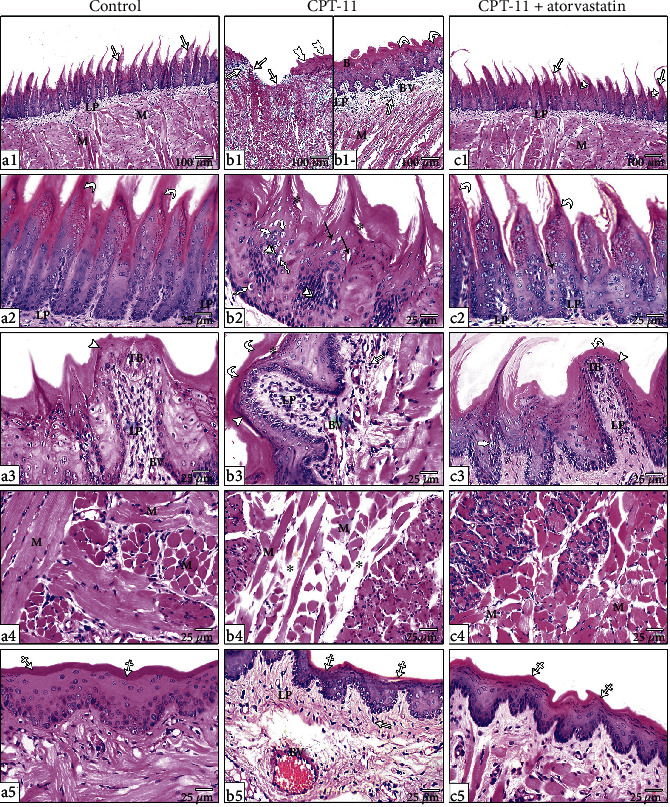
H&E-stained sections from the tongue of the control group (a), the dorsal surface showing regularly arranged conical shape filiform papillae (arrow) covered by stratified squamous keratinized epithelium with pointed tips (curved arrow) and underlying connective tissue cores continue with the lamina propria (LP). A characteristic fungiform papilla has broad top and narrow base with vascular connective tissue core (BV) and is covered by stratified squamous keratinized epithelium (arrowhead) contains barrel-shaped pale stained taste buds (TB). Bundles of lingual muscle fibers (M) run in various directions and surround by endomysium and perimysium that are continued with the connective tissue corium of both surfaces. The ventral surface is covered by smooth stratified squamous keratinized epithelium (crossed arrow) without papillae. CPT-11-treated group (b), the dorsal surface reveals the area of mucosal ulceration (arrow), other areas with short or absent filiform papillae (thick-tailed arrow), and blunt-ended tops (B) with inflammatory cell infiltration (double arrow) and congested blood vessels (BV) in the lamina propria (LP). The cells of stratified epithelium reveal vacuolation (zigzag arrow) and nuclear changes; chromatin margination with crescent formation (black arrow), pyknosis (thick white arrow), and chromatin condensation (double arrowheads). The fungiform papillae (arrowhead) show loss of taste buds and congested vascular core (LP) with inflammatory cell infiltration (double arrow). The papillae are covered by a thick layer of keratin (curved arrow) with areas of keratin separation (white asterisks). Disturbed organization of muscle fibers (M) with wide separation (black asterisks). The ventral surface shows thinning of the stratified epithelium with thin keratin layer (crossed arrow), and the lamina propria (LP) showed congested blood vessels (BV) with inflammatory cell infiltration (double arrow). CPT − 11 + atorvastatin treated group (c), the dorsal surface reveals normal orientation of the lingual papillae (arrow). Most of the filiform papillae are long with pointed tips and covered by stratified squamous keratinized epithelium (curved arrow), few papillae with loss of their tips (double arrowheads). Most of the cells are normal with few ones with marginated chromatin (black arrow) and few pyknotic nuclei (thick white arrow). Normal fungiform papillae (arrowhead) with taste bud (TB), normal thickness of keratin layer (curved arrow), and connective tissue core (LP) are observed. The organization of muscle layer (M) and the ventral surface (crossed arrow) closely resemble those of the control. (H&E a1, b1, c1 X100, a2 − 5, b2 − 5, c2 − 5 X400).

The H&E-stained tongue sections the control group (Figure 1(a) revealed that the dorsal surface was studded with conical-shaped filiform papillae). The papillae had a connective tissue (CT) core covered with stratified squamous keratinized epithelium with pointed tips. The papillae were regularly arranged and had a uniform shape and height (a1-2). The fungiform papillae had a distinctive mushroom shape with a broad top and a narrow base and were located between the filiform papillae, and taste buds were observed on their top surfaces. They were lined with stratified squamous keratinized epithelium (a3). The lamina propria contained CT with small-sized blood vessels. The main bulk of the tongue contained skeletal muscle fibers running in various directions. The muscle fibers were surrounded by the perimysium and endomysium that continued with the lamina propria CT (a4). The ventral surface of the tongue was covered with well-developed stratified squamous keratinized epithelium without lingual papillae (a5).

The CPT-11-treated group (Figure 1(b)) lost the normal organization of the dorsal surface papillae. Some of the filiform papillae were completely lost with multiple ulcerative areas (b1). Other papillae were short with blunted ends (b1). Areas of hyperkeratosis and keratin separation from the underlying epithelium were obvious (b2-3). Some of the epithelial cells showed vacuolar degeneration, while other cells revealed nuclear changes, including pyknosis, chromatin condensation, and chromatin margination with a crescent appearance (b2). The fungiform papillae were atrophied with ill-distinguished taste bud cells (b3). The skeletal muscle fibers were disorganized and widely separated (b4). The ventral surface showed a noticeable decrease in the thickness of the stratified squamous keratinized epithelium. The lamina propria revealed inflammatory cell infiltration and dilated, congested blood vessels (b5).

The CPT − 11 + atorvastatin − treated group (Figure 1(c)) showed restoration of normal lingual papillae organization (c1). The filiform papillae were covered with keratinized, stratified squamous epithelium with pointed ends. Most of the papillae had a uniform shape and height (c2). Most of the epithelium showed normal cytoplasm and nuclear appearances, apart from a few with nuclear chromatin margination and pyknotic nuclei (c3). The fungiform papillae were similar to that of the control group (c3). The CT had a normal arrangement of muscle fibers (c4). The ventral surface had average-sized, noninterrupted keratinized stratified squamous epithelium. The lamina propria displayed a disappearance of inflammatory cell infiltration and had normal-sized, noncongested blood vessels (c5).

### 3.2. Immunohistochemical Results

#### 3.2.1. Anti-Ki67

**Figure 2:**
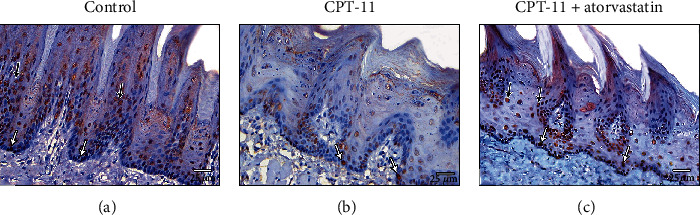
Ki-67 immunohistochemical staining. (a) (The control group) the dorsal surface shows frequent cells with positive immunoexpression in their nuclei in the basal (white arrow) and suprabasal layers (black arrow). (b) CPT-11-treated group reveals few positive cells mainly in the basal epithelial layer (white arrow). (c) CPT − 11 + atorvastatin treated group shows numerous immunostaining cells in the basal (white arrow) and suprabasal layers (black arrow) epithelial layer. (Ki-67 a-c X400).

Ki-67 immunohistochemical staining in the control groups revealed a positive reaction in the nuclei of the basal and suprabasal layers of the stratified epithelium on the dorsal surface of the tongue (Figure 2(a)). The CPT-11-treated group had few cells with positive reactions in their nuclei. The cells were mainly in the basal layer of epithelium (Figure 2(b)). The CPT − 11 + atorvastatin group revealed positive reactions in the nuclei of most of the basal and suprabasal epithelial cells (Figure 2(c)).

#### 3.2.2. Anti-Bcl2

**Figure 3:**
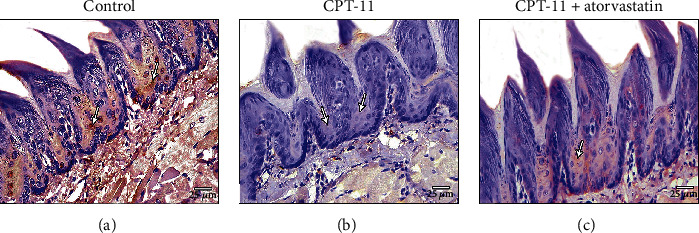
Bcl2 immunohistochemical staining. (a) The control group shows strong positive cytoplasmic immunoexpression to Bcl2 in most of the dorsal epithelial cells (arrow). (b) CPT-11-treated group reveals negative cytoplasmic reaction in most epithelial cells (arrow). (c) CPT − 11 + atorvastatin − treated group shows positive cytoplasmic immunoexpression with moderate density (arrow) in most of the epithelial cells. (Bcl2 a-c X400).

The control group had positive immunohistochemical reactions to Bcl2 in the cytoplasm of the stratified squamous epithelium (Figure 3(a)). The CPT-11-treated group had relatively negative Bcl2 immune expression in the cytoplasm of the stratified squamous cells (Figure 3(b)). The CPT − 11 + atorvastatin − treated group showed moderate positive immunohistochemical expression in the cytoplasm of the stratified squamous epithelium cells (Figure 3(c)).

#### 3.2.3. NF-*κ*B

**Figure 4:**
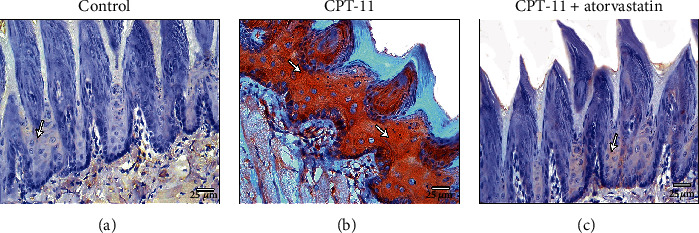
NF-*κ*B immunohistochemical staining. (a) The control group shows negative immunoexpression to NF-*κ*B in the cytoplasm of dorsal epithelial cells (arrow). (b) CPT-11-treated group reveals a strong positive cytoplasmic reaction in most epithelial cells (arrow). (c) CPT − 11 + atorvastatin treated group shows weak positive to negative cytoplasmic immunoexpression (arrow) in most of the epithelial cells. (NF-*Κ*b a-c X400).

NF-*κ*B immunohistochemical staining in the control groups revealed negative immune reactions in the cytoplasm of almost all epithelial cells (Figure 4(a)). The CPT-11-treated group had positive immune reactions in the cytoplasm of most cells of the different epithelial layers (Figure 4(b)). The CPT − 11 + atorvastatin − treated group had a few dispersed positive cells (Figure 4(c)).

### 3.3. Morphometric and Statistical Results

A nonsignificant change in the two main diameters of the filiform and the fungiform papillae was observed in the three-control subgroups. A statistically significant decrease was observed in the length and width of both filiform and fungiform papillae in the CPT-11-treated group compared to the control group. A nonsignificant change in the CPT − 11 + atorvastatin group in comparison to the control group was observed, but a significant increase was observed compared to the CPT-11-treated group (Tables 1 and 2).

A statistically significant decrease in the number of Ki-67 and Bcl2 positive cells in the CPT-11-treated group was observed, and a nonsignificant change in the CPT − 11 + atorvastatin group in comparison to the control group was observed (Table 3). A significant increase in the area percentage of the NF-*κ*B immunohistochemical results in the CPT-11-treated group was observed, but a nonsignificant change in the CPT − 11 + atorvastatin group was observed in comparison to the control group (Table 4).

### 3.4. Molecular Study Results

**Figure 5:**
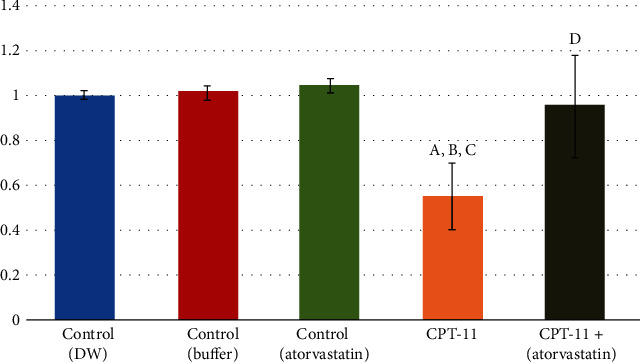
PCR level of KI-67 within the control and the experimental groups. (a) Comparison in relation to (-ve control DW) group. (b) Comparison in relation to (-ve control buffer) group. (c) comparison in relation to (+ve control atorvastatin) group. (d) Comparison in relation to (CPT-11) group.

**Figure 6:**
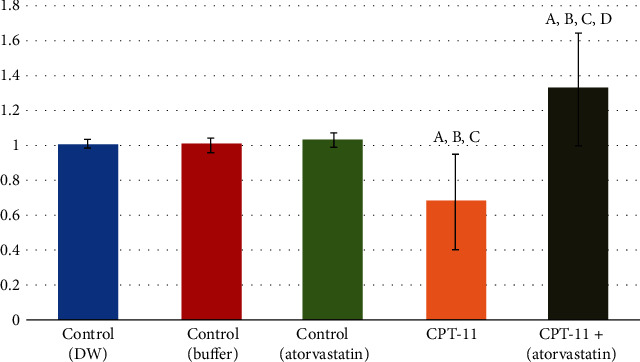
PCR level of BCL2 within the control and the experimental groups. (a) Comparison in relation to (-ve control DW) group. (b) Comparison in relation to (-ve control buffer) group. (c) Comparison in relation to (+ve control atorvastatin) group. (d) Comparison in relation to (CPT-11) group.

The immune-histochemical results were supported by the molecular gene expression results, since both Ki-67 and antiapoptotic Bcl-2 mRNA gene expression were significantly decreased (*P* < 0.001) in the tongue tissue of the CPT-11-treated groups. The gene expression levels increased in the CPT − 11 + atorvastatin to reach about the normal level of Ki-67 (0.95 ± 0.23) and even above normal levels of Bcl-2 (1.33 ± 0.32; Figures 5 and 6).

**Figure 7:**
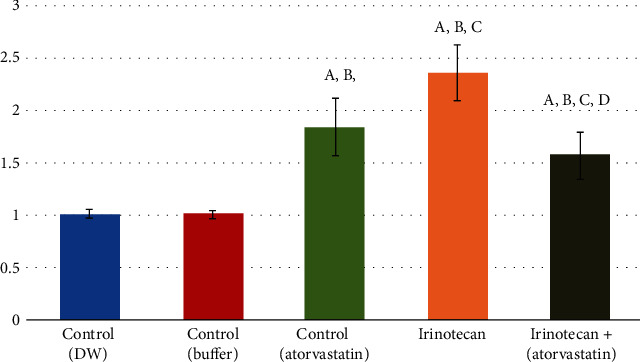
PCR level of NF-Kb level within the control and the experimental groups. (a) Comparison in relation to (-ve control DW) group. (b) Comparison in relation to (-ve control buffer) group. (c) Comparison in relation to (+ve control atorvastatin) group. (d) Comparison in relation to (CPT-11) group.

The gene expression changes in NF-*κ*B were consistent with the results of the histological examination of the tongue tissue samples. CPT-11 affects lingual cells by inducing marked inflammatory responses and increasing NF-*κ*B (2.36 ± 0.27, *P* < 0.001). In addition, atorvastatin increases NF-*κ*B mRNA levels, but not as much as CPT-11 (1.84 ± 0.28, *P* < 0.001); however, the coadministration of both drugs neutralized their individual effects on gene expression, which was still decreased, but above its normal levels (1.58 ± 0.23, *P* < 0.001; Figure 7).

**Figure 8:**
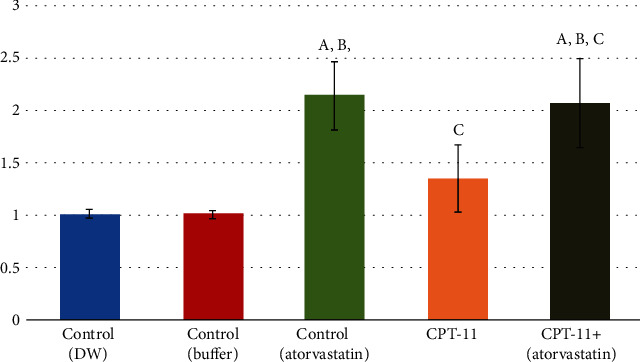
Nr-F2 level within the control and the experimental groups. (a) Comparison in relation to (-ve control DW) group. (b) Comparison in relation to (-ve control buffer) group. (c) Comparison in relation to (+ve control atorvastatin) group. (d) Comparison in relation to (CPT-11) group.

Nrf2 enhances antioxidant formation in response to oxidative stress through the Nrf2/ARE pathway. Atorvastatin caused highly significant increases in Nrf2 gene expression (2.14 ± 0.33, *P* < 0.001), while CPT-11 led to insignificant increases in its mRNA levels (1.35 ± 0.33, *P* = 0.061). CPT − 11 + atorvastatin resulted in highly significant increases in its gene expression (2.06 ± 0.41, *P* < 0.001; Figure 8).

## 4. Discussion

Oral mucositis is one of the most debilitating complications of chemotherapy and has proven clinical and financial implications. Despite the availability of multiple treatment services, there remains a need for the successful alleviation and prevention of chemotherapy-induced mucositis. In this study, the tongue was chosen to study the effects of CPT-11 on oral tissue since the tongue acts as a mirror of general personal health [22], especially the filiform papillae that can be damaged faster than other types of papillae [23].

The oral mucositis mechanism occurred as follows. Initially, chemotherapy causes cell damage and free radical production that activates a great number of transcription factors, such as NF-*κ*B. These transcription factors subsequently upregulate several genes that affect mucosal cell integrity, which results in basal epithelial cell death, apoptosis, tissue harm, and increased inflammatory factors, which increase cell death. Finally, the upregulation of proinflammatory cytokines occurs, including tumor necrosis factor- (TNF-) *α*, interleukin-1*β* (IL-1*β*), and interleukin-6 (IL-6), and the inflammatory processes are intensified, which causes ulcerations in the mucosa and speeds secondary infections [24, 25].

The CPT-11 treatment greatly disturbed the normal histological structure of the tongue. The dorsal surface revealed severely disrupted lingual papillae with some filiform papillae having blunted ends and others being atrophied. The results were confirmed by morphometric measurements of the two main diameters of the filiform papillae. A statistically significant reduction in the width and height of the papillae in comparison to the control group was observed. These results agree with Ibrahim and Elwan [12]. CPT-11 triggers its antitumor action by inflicting direct DNA damage and activating the innate immune response by initiating oxidative stress and releasing reactive oxygen species. CPT-11 also damages healthy cells, thus, causing gastrointestinal mucositis [26].

Our results showed an obvious thinning of the stratified squamous epithelium on both the dorsal and ventral surfaces of the tongue. These results were in line with Ibrahim and Elwan [12] and could be due to the reduction in cell proliferation, which was confirmed by a significant reduction in Ki-67 expression (a proliferation marker). Ki-67 expression was low in the immunohistochemical and gene expression results as well. This coincides with the results reported by de M Rêgo et al. [27] showing the antiproliferative role of CPT-11 that could explain the tongue's mucosal damage detected in this study. In the same context, a remarkable thickening of the keratin layer was observed, which was previously explained by the high sensitivity of taste buds to chemotherapy, taste bud damage, and the loss of sensory innervation which has been documented to have a role in disturbed keratinization and a loss of taste sensation [28]. In contrast, Kattaia et al. [29] attributed the hyperkeratinization of lingual papillae to the deficiency of the epidermal growth factor, which is involved in normal keratinization programming.

Vacuolar degeneration was observed in some stratified epithelium cells. The affected cells revealed vacuolated cytoplasm with condensed pyknotic nuclei. Our results were in harmony with Al Refai [30]. These degenerative changes could be due to the inhibition of topoisomerase I, which results in DNA damage and fragmentation. In contrast, the release of reactive oxygen species causes metabolism impairment in progenitor cells, DNA damage in the epithelial cells, mitosis inhibition, and increased apoptosis [31]. These results were confirmed by the immunohistochemical examination of Bcl2 immunostained sections and the quantitative real-time PCR of the Bcl2 mRNA, an antiapoptotic marker, which was significantly reduced in the CPT-11-treated group. Indeed, DNA injury triggered by the inhibition of topoisomerase I results in the stimulation of several pathways that are implicated in apoptotic death and inflammatory responses [32]. The decreased Bcl2 expression detected in the current study shows the action of CPT-11's antineoplastic effects by stimulating tumor cell apoptosis [33]. This could also affect nontumor cells, leading to the mucosal damage and mucositis observed in this study. These results were confirmed recently by Dai et al. [34], who showed that SN-38 (active metabolite of CPT-11) inhibits cell proliferation by arresting the cell cycle and stimulating apoptosis by blocking the antiapoptotic gene transcription of Bcl2.

In the present study, excess inflammatory cell infiltrations and dilated, congested blood vessels were obvious in the tongue CT of the CPT-11-treated group. Reactive oxygen species cause a series of biological events, resulting in the synthesis of different proinflammatory cytokines. These cytokines invade the epithelium, endothelium, and CT, causing tissue injury [35]. The damaging action of inflammatory mediators directly or indirectly increased vascular permeability and the absorption of cytotoxic drugs into the oral mucosa [36].

The histological results were confirmed by the significant increases in the expression of NF-*κ*B in the CPT-11-treated group at the mRNA level by qRT-PCR and the protein levels by immunohistochemical examination. NF-*κ*B is a main facilitator of proinflammatory gene initiation and functions [37], and it is also considered the main regulator in cellular proliferation, differentiation, angiogenesis, and apoptosis [38]. The NF-*κ*B signalling pathway includes a family of transcription factors that plays a role in immunity and inflammation [39]. NF-*κ*B is expressed in cells in response to various stressors, such as chemotherapy, to enhance cell survival. Many anticancer drugs, including CPT-11, enhance the gene expression of NF-*κ*B. This drug-induced activation is clinically undesirable as it may cause cells to develop a resistance to chemotherapeutic treatment [40]. Moreover, it is reported that the induction of the NF-*κ*B gene may trigger the induction of other inflammation genes [41], thus, causing inflammation as observed in the current study.

Surprisingly, the Nrf2 gene expression was increased in the CPT-11-treated animals. This result could be explained by the fact that Nrf2 is a transcription factor that enhances antioxidant production in response to oxidative stress through the Nrf2/ARE pathway, since CPT-11 induces oxidative stress [42]. Thus, increased Nrf2 gene expression could be a defense mechanism against CPT-11-induced oxidative stress. The Nrf2 antioxidant mechanism in response to oxidative stress could be explained as follows: Nrf2 is a member of the Cap'n'Collar transcription factor family. Its main function is to reestablish cellular redox homeostasis. The Nrf2 redox defense system mediates its effect through enzymes that are involved in sulfhydryl metabolism and iron homeostasis. NADPH is an essential coenzyme for the redox cycling mechanisms of the KEAP1/NRF2 pathway since NADPH coordinates a perfect reorganization of the cellular metabolism. This is helpful to resist many redox stressors and, consequently, to maintain normal cellular homeostasis [43]. The oxidative stress in this study had possible inducing factors, including the chemotherapeutic effect of CPT-11 [44] and the associated inflammatory reaction [45].

In this work, the CPT 11 + atorvastatin group had almost normal tongue architecture with preserved papillae and taste buds. These results are in line with the results of previous researchers who attributed the ability of statins to interfere with topoisomerase II function by inhibiting the action of the Ras-homologous GTPase Rac1, which is essential for the multiple cleavage process in DNA. A similar effect was shown in liver cells and cardiomyocytes after exposure to doxorubicin [46, 47]. In addition, Ziegler et al. [25] reported that the pretreatment of human keratinocytes in vitro with lovastatin reduced the DNA damage by doxorubicin by 75%. Statins decreased ATR/Chk1-regulated replicative stress, thus, preventing DNA damage, and mm preserving keratinocytes. Our histological results were in line with the expression of Bcl2 at the molecular and the immunohistochemical levels. Our results revealed the upregulation of the Bcl2 gene and protein levels in the atorvastatin-treated group, which could be due to the cytoprotective effect of statins caused by the inhibition of apoptotic cell death. This study confirms the upregulating effect of the statin drug family on Bcl2 gene expression as reported by Franke et al. [48].

Our results revealed a significant decrease in the expression of NF-*κ*B mRNA and proteins in the atorvastatin-treated group. The anti-inflammatory property of statins was shown to be due to their inhibitory effects on inducible nitric oxide synthetases (iNOS), which are commonly utilized by cells as proinflammatory cytokines [49]. In addition, atorvastatin was reported to decrease TNF/IFN-stimulated iNOS expression in the endothelium cells of the aorta [50] and at the molecular level, as reported recently by Wang et al. [51].

Our results also revealed increased Nrf2 gene expression in the CPT − 11 + atorvastatin group. This result confirmed the stimulant effect of atorvastatin on the gene expression of Nrf2 as a marker of antioxidant defense and provided evidence of its potential protective effects against oxidative stress damage by oxidative stress induction caused by various inducers, including chemotherapy. The induction of Nrf2 gene expression by statins was also reported previously by Ihoriya et al. [52] and recently by Bao et al. [53], who reported that statins had an antioxidant effect by suppressing the formation of oxygen free radicals by inhibiting the activation of the NADPH oxidase complex, which is responsible for superoxide generation [54]. In addition to its cholesterol-lowering action, it has several cholesterol-independent effects that are valuable for human health. These pleiotropic effects include antioxidant properties [55], anti-inflammatory effects, immunomodulatory effects [56], and endothelial cell-defending actions through the upregulation of endothelial nitric oxide synthase (eNOS) [57].

## 5. Conclusions

In conclusion, the current study revealed prominent rat mucosal damage in the form of lingual mucositis that is induced by CPT-11 chemotherapy, an important alternative for patients with mucositis. The mechanism of CPT-11-induced mucosal damage could be related to chronic inflammation, oxidative stress, and an imbalance of the apoptotic and antiapoptotic pathways. These results were confirmed at the histological and molecular levels. Atorvastatin could play a protective role against CPT-11 mucosal damage by inducing an antiapoptotic gene and modulating inflammatory and antioxidant gene expressions.

## Figures and Tables

**Table 1:** Morphometric study of the length and width of filiform papillae within the control and the experimental groups.

	Study groups					Test of significance
	Group Ia (-ve control DW)	Group Ib (-ve control buffer)	Group Ic (+ve control atorvastatin)	Group 11 (CPT-11-treated)	Group III (CPT-11 + atorvastatin)	
Mean length (*μ*m)	310.26 ± 44.52	307.22 ± 45.35	312.03 ± 39.52	166.72 ± 20.18	302.22 ± 32.44	*F* = 85.471 *P* < 0.001^∗∗^
*P*_1_		0.782	0.848	<0.001∗∗	0.425	
*P*_2_			0.593	<0.001∗∗	0.580	
*P*_3_				<0.001∗∗	0.276	
*P*_4_					<0.001∗∗	

Mean width (*μ*m)	107.75 ± 32.65	110.75 ± 30.45	105.75 ± 30.44	94.88 ± 15.65	102.43 ± 29.5	*F* = 57.719 *P* < 0.001^∗∗^
*P*_1_		0.430	0.557	0.005∗	0.218	
*P*_2_			0.229	<0.001∗∗	0.078	
*P*_3_				0.021∗	0.444	
*P*_4_					0.046∗	

SD: standard deviation, F for ANOVA test. ∗Statistically significant if *P* ≤ 0.05. ∗∗Highly statistically significant result if *P* ≤ 0.001. *P*_1_: comparison in relation to (-ve control DW) group. *P*_2_: comparison in relation to (-ve control buffer) group. *P*_3_: comparison in relation to (+ve control Atorvastatin) group. *P*_4_: comparison in relation to (CPT-11) group.

**Table 2:** Morphometric study of the length and width of fungiform papillae within the control and the experimental groups.

	Study groups					Test of significance
	Group Ia (-ve control DW)	Group Ib (-ve control buffer)	Group Ic (+ve control atorvastatin)	Group 11 (CPT-11-treated)	Group III (CPT-11 + atorvastatin)	
Mean length (*μ*m)	224.15 ± 21.79	218.15 ± 23.59	220.15 ± 19.55	188.51 ± 36.4	219.22 ± 20.33	*F* = 32.209 *P* < 0.001^∗∗^
*P*_1_		0.404	0.675	<0.001∗∗	0.529	
*P*_2_			0.850	<0.001∗∗	0.918	
*P*_3_				<0.001∗∗	0.984	
*P*_4_					<0.001∗∗	

Mean width (*μ*m)	175.61 ± 24.35	177.55 ± 20.35	174.5 ± 22.25	110.93 ± 31.11	169.24 ± 18.41	*F* = 81.456 *P* < 0.001^∗∗^
*P*_1_		0.715	0.889	<0.001∗∗	0.320	
*P*_2_			0.654	<0.001∗∗	0.274	
*P*_3_				<0.001∗∗	0.395	
*P*_4_					< 0.001∗∗	

**Table 3:** Number of anti-Ki 67 and anti-Bcl2 immune-stained cells within the control and the experimental groups.

	Study groups					Test of significance
	Group Ia (-ve control DW)	Group Ib (-ve control buffer)	Group Ic (+ve control atorvastatin)	Group 11 (CPT-11-treated)	Group III (CPT-11 + atorvastatin)	
Anti-Ki 67	54.12 ± 2.3	50.22 ± 1.8	52.15 ± 2.4	22.3 ± 1.9	49.33 ± 2.1	*F* = 27.410 *P* < 0.001^∗∗^
*P*_1_		0.667	0.882	<0.001∗∗	0.395	
*P*_2_			0.836	<0.001∗∗	0.964	
*P*_3_				<0.001∗∗	0.703	
*P*_4_					<0.001∗∗	

Anti-Bcl2	15.2 ± 2.5	16.2 ± 1.4	15.5 ± 2.2	2.6 ± 2.1	13.8 ± 1.9	*F* = 23.257 *P* < 0.001^∗∗^
*P*_1_		0.626	0.918	<0.001∗∗	0.472	
*P*_2_			0.782	<0.001∗∗	0.260	
*P*_3_				<0.001∗∗	0.508	
*P*_4_					<0.001∗∗	

**Table 4:** The area percentage of positive anti-NF*κ*B immune reactions within the control and the experimental groups.

	Study groups					Test of significance
	Group Ia (-ve control DW)	Group Ib (-ve control buffer)	Group Ic (+ve control atorvastatin)	Group 11 (CPT-11-treated)	Group III (CPT-11 + atorvastatin)	
	2.5 ± 1.9	2.2 ± 1.5	3.3 ± 1.5	18.4 ± 2.8	4.2 ± 2.1	KW = 17.549 *P* < 0.001^∗∗^
*P*_1_		0.527	0.460	<0.001∗∗	0.184	
*P*_2_			0.255	<0.001∗∗	0.122	
*P*_3_				<0.001∗∗	0.288	
*P*_4_					<0.001∗∗	

SD: standard deviation, KW for Kruskal Wallis test. ∗Statistically significant if *P* ≤ 0.05. ∗∗Highly statistically significant result if *P* ≤ 0.001. *P*_1_: comparison in relation to (-ve control DW) group. *P*_2_: comparison in relation to (-ve control buffer) group. *P*_3_: comparison in relation to (+ve control atorvastatin) group. *P*_4_: comparison in relation to (CPT-11) group.

## Data Availability

The data that support the findings of this study are available from the corresponding author upon reasonable request.

## Supplementary Materials

Supplementary Materials Supplementary material 1: description of the primer sequences used in this study.
