# Supplementary material for: Study of the Possible Alleviated Role of Atorvastatin on Irinotecan-Induced Lingual Mucosal Damage: Histological and Molecular Study
Source: Oxid Med Cell Longev. 2021 Sep 30;2021:9690047. doi: 10.1155/2021/9690047 (PMC8497104; doi:10.1155/2021/9690047)
Supplement: Supplementary Materials — Supplementary material 1: description of the primer sequences used in this study. [file 9690047.f1.docx]

The primer sequences used were rat Ki-67: forward, 5ʹ- ATT TCA GTT CCG CCA ATC C-3ʹ; reverse, 5ʹ- GGC TTC CGT CTT CAT ACC TAA A-3ʹ[19], rat Bcl-2: forward, 5ʹ- TGT GGA TGA CTG ACT ACC TGA ACC -3ʹ; reverse, 5ʹ- -CAG CCA GGA GAA ATC AAA CAG AGG -3ʹ [20], rat NF-κB: forward, 5ʹ- CTA GCT AGC TAC GGC ATC GAT CG -3ʹ; reverse 5ʹ- CGT AGG AGT CGA TCG ATA TAG CTA CG -3ʹ [21], rat Nrf2: forward, 5ʹ- CAC ATC CAG ACA GAC ACC AGT -3ʹ; reverse 5ʹ- CTA CAA ATG GGA ATG TCT CTG C -3ʹ [20].

Rat β-actin primers sequences were 5ʹ- AAG ATC CTG ACC GAG CGT GG-3ʹ (Forward) and 5ʹ- CAG CAC TGT GTT GGC ATA GAG G -3ʹ (Reverse) as an internal control gene. The expression of the analyzed genes was normalized to that of the β-actin as an internal control gene using the comparative ∆∆CT method [20].
